# Case Report: A case of ectopic tooth with fungal infection in the maxillary sinus

**DOI:** 10.3389/fsurg.2025.1564859

**Published:** 2025-04-17

**Authors:** Zhao Shumin, Zhao Qiuliang

**Affiliations:** Department of Otolaryngology, Head and Neck Surgery, Central Hospital Affiliated of Shandong First Medical University (Jinan Central Hospital), Jinan, China

**Keywords:** fungal infection, sinusitis, excision, maxillary sinus, heterotopic teeth

## Abstract

This paper reports a case of a maxillary sinus ectopic tooth with fungal infection admitted to the Department of Otorhinolaryngology and Head and Neck Surgery, Affiliated Central Hospital of Shandong First Medical University. The patient, a 74-year-old female, was admitted due to a sensation of “head tightness” and underwent endoscopic resection of the left maxillary sinus open lesion and anterior lacrimal recess approach for maxillary sinus ectopic tooth, with good postoperative results.

## 1 Clinical data

**Figure 1:**
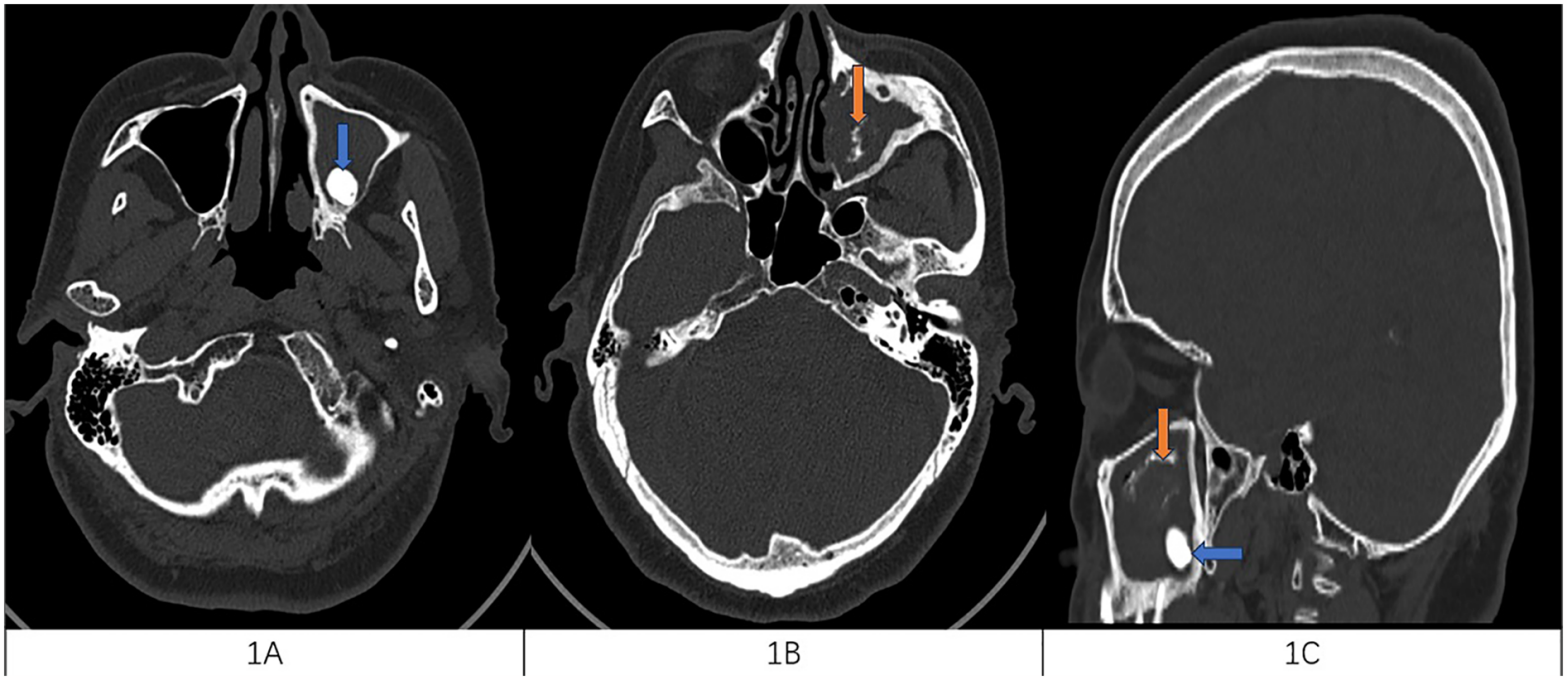
**(A)** Horizontal CT scan of the sinuses with a blue arrow on the left side of the maxillary sinus with high-density mass considering the ectopic tooth. **(B)** The orange arrow on the horizontal sinus CT shows a high-density cord image of the left maxillary sinus, considering fungal infection. **(C)** Both lesions were present on the CT disfigurement of the sinuses orange for fungal infection and blue for ectopic teeth.

**Figure 2:**
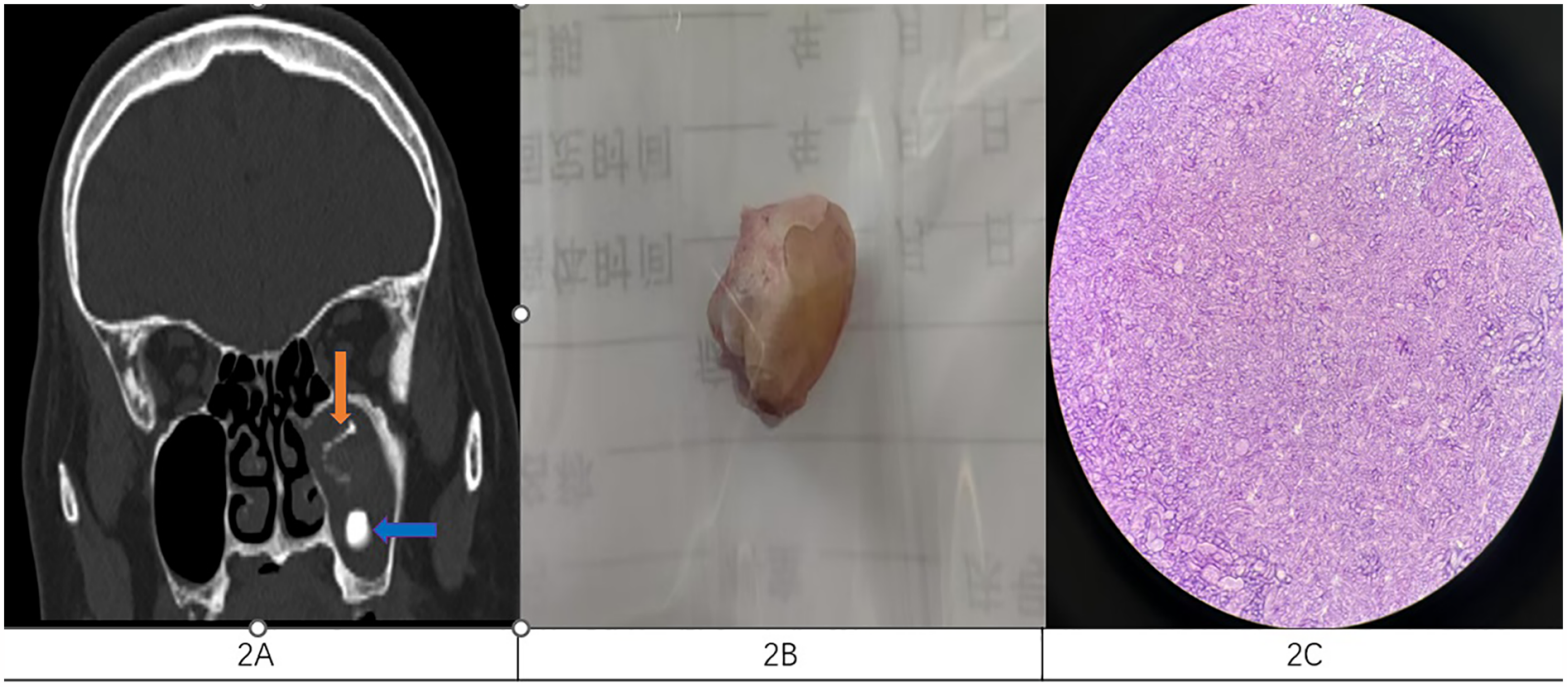
**(A)** Coronal CT scan of the sinuses showed the presence of both lesions orange for fungal infection and blue for ectopic teeth. **(B)** The ectopic tooth was removed intraoperatively. **(C)** Pathological smears showing fungi.

A 74-year-old female patient presented with a 1-month history of headache accompanied by an abnormal odor in the left nasal cavity, mainly in the occipital area. There was no significant symptoms of nasal congestion or rhinorrhea. She was admitted to the Department of Neurology of our hospital, and a completed cranial CT examination revealed that the ectopic tooth in the left maxillary sinus was complicated with fungal infection (see Figures 1A–C, 2A), and no acute brain lesions were found. She was subsequently referred to our department for surgical treatment. Preoperative examination ruled out surgical contraindications. During the surgery, the left uncinate process was removed, and the maxillary sinus was opened. A large amount of caseous matter and purulent secretions were found in the sinus cavity. The maxillary sinus opening was enlarged, the fungal mass and purulent secretions were removed, and caseous matter was retained for routine pathology examination. It was found that the ectopic tooth was located posteriorly outside the maxillary sinus cavity, making it difficult to extract through the middle nasal meatus. Therefore, an approach through the left inferior nasal meatus and the prelacrimal recess was considered to access the maxillary sinus cavity. The specific procedure was as follows: A vertical incision was made along the anterior edge of the left inferior turbinate at the mucocutaneous junction, extending from top to bottom to the nasal floor, cutting through the mucoperiosteum to the bone. The mucoperiosteum of the lateral nasal wall was separated from front to back, and the bone around the nasolacrimal duct was removed to fully free the nasolacrimal duct. The bone of the medial wall of the left maxillary sinus was removed to create a surgical pathway from the front of the nasolacrimal duct to the maxillary sinus, providing adequate exposure. An ectopic tooth was then chiseled out and removed (Figure 2B), and the root of the lesion was thoroughly coagulated using an electric knife. The mucosa was repositioned and sutured, and the surgery was completed successfully. The postoperative pathological examination of the caseous material revealed fungal masses (Figure 2C). Following the surgery, the patient's clinical symptoms resolved completely. The patient is currently under postoperative follow-up for nasal endoscopy.

## 2 Discussion

An ectopic tooth is when there is a tooth defect in the dentition and the tooth erupts outside the tooth-containing area. Ectopic teeth are common in the dental arch region, and the probability of occurrence in non-dental areas is relatively low, such as mandibular condyle, coronal process, orbit, upper palate, maxillary sinus, nasal cavity, and septum (1).

Although the etiology of ectopic teeth has not been determined, it is thought that it may be related to the abnormal interaction of oral epithelial and mesenchymal tissues during tooth formation (2, 3). Although heterotopic maxillary sinus teeth have been reported in the literature, they are not common (4). It is worth noting that very few cases have been reported in ENT departments. The incidence is 0.1%–1%, and most patients are male (5). The etiology of maxillary sinus ectopic teeth is not completely clear, but it may be related to the following factors. (1) Abnormal development: During the development of teeth, the tooth embryo may be displaced due to genetic or environmental factors, resulting in the formation of teeth in the maxillary sinus. (2) Trauma: Facial trauma may displace the tooth embryo and enter the maxillary sinus. (3) Infection or inflammation: Infection or inflammation of the maxillary sinus or surrounding tissues may affect the normal development and position of the tooth embryo. (4) Cysts or tumors: The growth of cysts or tumors may press on the tooth embryo, causing it to shift to an abnormal position. (5) Surgical complications: Oral or maxillary sinus surgery may accidentally lead to tooth embryo displacement (6). Ectopic teeth may lead to complications if not treated in time. A sinus fungal infection is an inflammation of the sinuses caused by fungi and is commonly seen in people with weakened immune systems. Common pathogens include *Aspergillus* and *Mucor*. Symptoms include nasal congestion, facial pain or pressure, nasal discharge (which may be bloody), decreased sense of smell, headache, and fever (invasive infection). The diagnosis mainly depends on imaging examination, and the confirmed diagnosis requires pathological examination. CT findings may include the following. (1) High-density imaging: Fungal spheres or allergic fungal sinusitis often show high-density imaging, which may be accompanied by calcification. (2) Cloudy sinus cavity: The affected sinus cavity is cloudy with uneven density. (3) Bone changes: Chronic infections may lead to thickening or destruction of bone. (4) Unilateral involvement: Fungal globules often occur unilaterally, mostly in maxillary sinus fungal sinusitis. The main pathological manifestations are fungal masses or allergic mucus, which may be accompanied by necrosis and inflammation. The main treatment option is surgery. Ectopic teeth may lead to complications if not treated in time. In this case, ectopic tooth with fungal infection was rare. Due to the atypical symptoms of the patient, the presence of ectopic teeth was found by cranial CT. If the ectopic tooth and infection are not treated in time, it may lead to further spread of infection, such as secondary orbital infection and bone destruction. In terms of treatment, the complete removal of ectopic teeth and infected tissues is of great significance for the recovery of the disease and the reduction of postoperative recurrence. Clinically, appropriate surgical methods should be selected according to the implant site of heterotopic teeth in the sinus cavity, and minimally invasive concepts should be implemented to improve patients' subjective feelings and reduce the occurrence of surgical complications (7). Previous open operations such as lateral rhinotomy or traditional Caldwell–Luc surgery cause greater damage to patients and are prone to lead to facial deformities in the later stage, resulting in reduced quality of life. In this case, the natural sinus approach of the maxillary sinus was used first to clear the infected lesions, and then the inferior nasal passage anterior lacrimal recess approach was used to remove the ectopic tooth in the maxillary sinus cavity today. Compared with traditional surgical methods, the anterior lacrimal recess approach is less invasive in removing the concealed lesions in the sinus, which reduces the bone defect in the anterior maxillary sinus wall, maintains a clear surgical field, and reduces surgical trauma. The probability of complications such as lip and face numbness caused by infraorbital vascular nerve bundle injury was avoided successfully, the length of hospital stay was shortened, and the quality of life of patients was improved.

## Data Availability

The original contributions presented in the study are included in the article/Supplementary Material; further inquiries can be directed to the corresponding author.
